# Supplementary material for: Characterizing a species-rich and understudied tropical insect fauna using DNA barcoding
Source: Gigascience. 2026 Mar 17;15:giag028. doi: 10.1093/gigascience/giag028 (PMC13108253; doi:10.1093/gigascience/giag028)
Supplement: giag028_Authors_Response_To_Reviewer_Comments_original_submission [file giag028_authors_response_to_reviewer_comments_original_submission.pdf]

Author's Response To Reviewer Comments

To all reviewers, thank you very much for your positive comments. We have addressed all of your recommended changes and feel that the manuscript is improved as a result. We respond to all specific reviewer comments below:

Reviewer #1:

The manuscript "Characterising a species-rich and understudied tropical insect fauna using DNA Barcoding" by Hemprich-Bennett and co-authors provides DNA barcodes from 95,996 individuals sampled in Ghana using various trap systems. In total, 10,120 unique BINs were identified, including 4,939 that were newly generated. Most sampled taxa were Diptera, Coleoptera, and Lepidoptera. In addition, the authors compared the determined BINs with already published data at BOLD, revealing the greatest overlap in BIN sharing with South Africa.
In my eyes, the topic of this manuscript is interesting and for suitable for a publication in "GigaScience" that is focusing on "big data" research. The amount of new sequence data for arthropods, in particular insects, is awesome and represents an important step to assess the (molecular) biodiversity, or better species diversity, of a super diverse region which has hardly been studied so far. The authors use state-of-the-art methods to analyze their data including the BOLD database and BIN approach. However, there are some points that should be added or discussed in a broader context (see below). In addition, please find some specific comments made via sticky notes on the PDF file of the manuscript.

- I feel that the authors should provide some more references on various topics, especially in the introduction but discussion, too.
We have added numerous additional references throughout the introduction and discussion
- It would be nice to present some maps, photos of the collection sites, the sampling devices as well as the samples themselves as part of the main manuscript, documenting the efforts that were taken.
We have included maps and photos of traps in the study sites in supplementary figures 1-6
- A BIN does per se not represent a species, because the variability of the DNA barcode fragment and mitochondrial DNA in general can be affected by various effects, e.g., incomplete lineage sorting, Wolbachia infections (especially true for arthropods), phylogeographic events, hybridization, and others. As consequence, BIN sharing and splitting can be observed - and in fact such effects are more often found than expected. It is fully clear that such analysis cannot be done for the given dataset, but a discussion of these effects is important and has been lacking thus far.
We have now added a brief discussion of this to the introduction (lines 86-93, “BINs are not 1:1 equivalents for species, as within-species variability in the standard DNA barcode segment is not uniform across broader taxonomic groups, and phenomena such as Wolbachia infections [19], between-species hybridisation, and errors in data uploaded to BOLD [20] can lead to single BINs containing multiple species and vice versa. The addition of new sequencing data to BOLD can also prompt the revision of BIN assignments, making any individual assignment somewhat provisional [21].”)
- What happened with the vouchers and DNA extracts? It is obvious that the collected specimens will include a high number of undescribed species, therefore the deposition of the voucher specimens is highly important.
Introduction lines 117-121: “Voucher specimens are currently stored at the Barcode of Life Database site in Guelph, Canada, and can be requested for further analyses; in accordance with the Nagoya Protocol Material Transfer Agreement, all specimens and any remaining DNA extract will be returned to University of Ghana upon request.”
- In my eyes it would be interesting to provide a summary of the lengths of the barcodes that were studied. How many barcodes were complete with a length of 658 base pairs? How many were about 300 bp etc.? I think such analysis can be easily done and visualized.
We’ve added a sentence to the results and a plot to the SI "Of the 95,996 samples analysed, sequences were obtained from 81,518 (mean sequence length 653.4bp, s.d 14.4bp, see Supplementary Figure 7).”
- Lines 86-87: “BINs are clusters of highly similar DNA sequences and so can act as a species-proxy when a species has yet to be formally described” BINs are not written in stone and may change due the addition

of new species/sequences. This important fact must be mentioned.
We have included this in the new section of the introduction mentioned above (lines 86-93) “BINs are clusters of highly similar DNA sequences and so can act as a species-proxy when a species has yet to be formally described. BINs are not 1:1 equivalents for species, as within-species variability in the standard DNA barcode segment is not uniform across broader taxonomic groups, and phenomena such as _Wolbachia_ infections [19], between-species hybridisation, and errors in data uploaded to BOLD [20] can lead to single BINs containing multiple species and _vice versa_. The addition of new sequencing data to BOLD can also prompt the revision of BIN assignments, making any individual assignment somewhat provisional [21].”
- Lines 221-222: “The percentage constitution of each taxonomic order of samples sequenced per trap type. E.g. 57.85% of all samples sequenced from CDC traps were Dipterans.” It would be good not only to provide percentage values but total values as well.
This was already included in the SI (tables 1 and 2) but admittedly it was previously unclear due to an error in the tables’ legends that is now corrected: the tables contain the number of samples of a given taxa in each trap type, not the number of BINs of a given taxa as previously stated.
- Line 381: “more insFect individuals” typo?
Yes, now rectified
- Lines 394-396: “While BINs are an invaluable first step in describing biodiversity, we encourage efforts towards the formal taxonomic description of the many unnamed taxa within this understudied arthropod assemblage.” This can be only done when the voucher are still existing. Is this the case? Where are they deposited? Guelph? Or at a museum (favoured)?
We’ve now added a sentence in the introduction, lines 117-121 "Voucher specimens are currently stored at the Centre for Biodiversity Genomics site in Guelph, Canada, and are available for further analyses; in accordance with the Nagoya Protocol Material Transfer Agreement, all specimens and any remaining DNA extract will be returned to University of Ghana upon request."

Reviewer #2: General Comments:

This manuscript presents an impressive and highly valuable study that significantly advances our understanding of tropical arthropod diversity in West Africa. The sampling effort is extraordinary (nearly 100,000 individuals sequenced), and the dataset generated more than doubles the number of Barcode Index Numbers (BINs) publicly available for the region. The study is well-designed, employing multiple complementary trap types to capture diverse components of the arthropod community. The analyses are generally robust and appropriate for the research questions. The public release of this large dataset is a major contribution that will undoubtedly stimulate further taxonomic and ecological research in understudied tropical regions. The manuscript is clearly written and well-structured. I am generally in favour of acceptance after minor revisions.

Specific Comments and Suggestions for Revision:
- The manuscript would benefit from including representative photographs of each of the five trap types (Malaise, yellow pan, pitfall, Heath, CDC) as deployed in the field. This is particularly helpful for readers less familiar with entomological methods. Given potential space constraints in the main text, I recommend including these as a Supplementary Figure (e.g., a panel of five photos with concise captions). Please cite this figure in the Methods (Sampling) section.
We’ve now included photos of each of these trap types in the Supplementary Information, and refer to them in the Methods (lines 145-149) “At 10 m, 20 m, 30 m, and 40 m, we placed a yellow pan trap and a pitfall trap (Supplementary Figure 4), both filled with soapy water on alternating sides of the transect line. We set a Heath trap (Supplementary Figure 5) at 50 m and a CO_2-baited Center for Disease Control (CDC) trap (Supplementary Figure 6) at the 100 m point to avoid interference with other traps.”
- The NMDS and PERMANOVA results convincingly show differences among trap types. However, the sequencing effort (and thus sample size) varied greatly among traps (e.g., Heath: 65,293 samples vs. CDC: 3,039 samples). Could the authors please clarify if the Bray-Curtis dissimilarity matrices used in these analyses were calculated on standardized or rarefied data to account for this large disparity in sample size? A brief note in the Methods (Data analyses) or figure legend would assure readers that the observed patterns are not primarily an artefact of sampling intensity.

We used the raw data as in this case the sampling effort was quite standard across trap-types. The disparity in number of samples per trap-type that the reviewer comments on is a function of how productive a given trap type is (Heath traps just catch far more insects than CDC traps) and so it is not informative to try and control for this difference. We now clarify the data source in the Methods section (line 187-188, "Taxonomic groups were only included in the analyses if they contained a minimum of 10 samples, with the raw abundance of each taxonomic group in a trap type being used.")
- The finding of significantly higher diurnal catches (individuals and BINs) in Malaise traps is interesting. The discussion briefly mentions variance in thermal conditions. Could the authors expand the Discussion (Diurnal activity patterns) to include other potential ecological or methodological explanations? For example, might this reflect true peaks in flight activity for dominant taxa (Diptera, Hymenoptera), or could it be influenced by trap visibility or wind patterns differing between day and night? A sentence or two of speculation would enrich the interpretation.
We now include two new sentences (lines 413-418) "Wong and Didham [50] previously found overall global insect activity patterns to be higher at night than in the day, but the effect was influenced by both insect community composition and habitat type. Activity patterns were higher during the day in grasslands, savannahs and forests, habitat types that are somewhat analogous to our matrix of grassland, cropland and forest fragments, and all habitats where there is a strong variation between daytime and nighttime temperatures."
- The authors transparently note that only 34 of 117 Malaise lots were fully sequenced and that spiders were removed from some analyses. In the Discussion, please add a short statement evaluating how these practical limitations might have influenced the key conclusions regarding trap complementarity and overall community completeness. For instance, does the high rate of BIN accumulation in Malaise traps (Supplementary Figure 6) suggest that sequencing the remaining lots might have yielded many additional unique BINs, potentially altering the estimated contribution of this trap type?
We now include a sentence acknowledging this limitation (lines 385-387) "Given that malaise trap samples showed the highest rate of BIN accumulation (Supplementary Figure 11) but unfortunately many malaise trap lots were unable to be sequenced, this will have likely reduced the overall contribution of this trap type."
- Line 381: There is a typo: "more insFect individuals" should be "more insect individuals".
Thanks, now corrected
- Figure 2 & 3 Citations in Text: The in-text citations for Figures 2 and 3 (e.g., lines 239, 274-277) are currently embedded in the legend descriptions copied from the PDF. These should be simplified to standard figure calls (e.g., "(Figure 2)", "(Figure 3A, B)") and the legend text removed from the main manuscript body.
We've now removed the formatting linking the in-text citations to the objects. Regarding the legend texts being included in the main manuscript body, this was done to comply with Gigascience's author guidelines: "Figure titles (max 15 words) and legends (max 300 words) should be provided in the main manuscript, not in the graphic file."

Reviewer #3:

This paper describes a massive DNA barcoding project of arthropods in Ghana, West Africa with a dataset of 95,996 individuals and 10,120 BINs (Barcode Index Numbers). The publication is a major contribution to characterizing biodiversity of tropical insects in a poorly studied area, answering methodological questions concerning trap complementarity and temporal activity, and is also an invaluable resource to the public. The research is well structured, analyses are favorable and the manuscript is well written. I recommend acceptance after minor revisions to address a few clarifications and technical points.

- The manuscript acknowledges that only a subset of individuals was sequenced due to logistical constraints, and for Heath traps, selection was based on wet mass. While the authors argue that sub-sorting aimed to maximize diversity, this could still introduce biases in abundance estimates and BIN accumulation curves. Please include a brief discussion of how this sub-sampling might affect the conclusions (e.g., richness estimates, trap comparisons) and consider adding a sensitivity analysis in the supplement if feasible.
A sensitivity analysis is not feasible as we do not have any data in which comparable lots were/weren't subsampled. We now include an expanded acknowledgement of this limitation in lines 383-387

“Nevertheless, cryptic taxa may be under-detected due to this limitation, potentially impacting rates of BIN accumulation or trap complementarity. Given that malaise trap samples showed the highest rate of BIN accumulation (Supplementary Figure 11) but unfortunately many malaise trap lots were unable to be sequenced, this will have likely reduced the overall contribution of this trap type.”
- The finding that South Africa shares the most BINs with Ghana despite geographic distance is interesting and attributed to sampling effort. However, the regression model explains only 3% of variance (R2=0.03), suggesting other factors may be at play. Please discuss potential biogeographic or ecological reasons (e.g., similar habitats, historical connectivity) that might contribute to this pattern, even if sampling effort is the dominant driver.
We now include a sentence on this in the discussion (lines 339-341), “The model had a low R2 (0.03), indicating that other variables are likely important for the number of shared taxa, such as the country’s climate, habitat types, or trap types most used in sampling.”
- The use of BINs as a species proxy is appropriate for this study, but the manuscript should briefly acknowledge known limitations (e.g., BINs may over- or under-split species, particularly in poorly studied taxa). A sentence or two in the Discussion would suffice, noting that BINs are a pragmatic tool for biodiversity assessment but not a replacement for formal taxonomy.
We have now included a section in the introduction addressing this (lines 86-93) “BINs are clusters of highly similar DNA sequences and so can act as a species-proxy when a species has yet to be formally described. BINs are not 1:1 equivalents for species, as within-species variability in the standard DNA barcode segment is not uniform across broader taxonomic groups, and phenomena such as _Wolbachia_ infections [19], between-species hybridisation, and errors in data uploaded to BOLD [20] can lead to single BINs containing multiple species and _vice versa_. The addition of new sequencing data to BOLD can also prompt the revision of BIN assignments, making any individual assignment somewhat provisional [21].”
- Line 381: "insFect" should be "insect".
Now corrected
- Table 1 and Table 2 are well-presented, but consider adding a footnote explaining that "BINs unique to trap type" means not found in other trap types in this study.
Now updated
- Line 140: Specify the soap concentration used in pan and pitfall traps.
We added a few drops of dish washing liquid to break the surface tension, giving a ratio of approximately 1 tsp per gallon (https://www.frontiersin.org/journals/ecology-and-evolution/articles/10.3389/fevo.2020.579193/full#B72) but didn’t state this in the manuscript as this was not a precise concentration. This is a well-established protocol, and as we stored insects in 95% ethanol after removal from the trap, does not affect the DNA barcoding protocol
- Line 150: Clarify how "wet mass" was measured (precision, handling protocol).
We now specify this on lines 158-161: “The number of arthropods selected for sequencing per Lot was in proportion to their wet mass, determined as the weight after filtering off ethanol through Nitex mesh. Following a brief visual inspection, samples were selected to maximise the number of morphospecies and in approximate proportion to Lot contents.”
- Line 156: Mention the success rate of PCR and sequencing (how many samples failed?).
We now mention this in the beginning of the results: “Of the 95,996 samples analysed, sequences were obtained from 81,518 (mean sequence length 653.4bp, s.d 14.4bp, see Supplementary Figure 7).”
- Line 360-379: The section on "Taxa of potential human importance" is interesting but could be strengthened by relating findings to local agricultural or health contexts. For example, what do the low numbers of crop pests or disease vectors imply for local management?
We are hesitant to make any strong statements on this as we don’t know how likely pests and vectors are to be captured relative to their local abundance and we have little data available on the damage that these species are causing in the area (except for a paper on the fall army worm). For us the main take-home is that when using non-targeted trapping methods these taxa are found to be a small fraction of the arthropod community. We highlight this to show how our own biases might lead to a misinterpretation of the relative abundance of a species that we are interested in, but in our opinion, there aren’t any explicit recommendations etc that can be made from our sampling for local management.
- Line 390-396: The conclusion could briefly highlight future directions, e.g., integrating morphological taxonomy with BINs, or using this dataset for metabarcoding studies.
We mention that the voucher specimens can be used for formal description of the BINs released here. We are hesitant to lengthen the short paragraph by adding discussion of metabarcoding use-cases, as we discuss in the introduction (lines 112-115) that this dataset was primarily collected for use in

metabarcoding research. “Our insect sampling and DNA barcoding campaign primarily took place to make a reference library for use in an ongoing dietary metabarcoding project investigating the position the mosquito malarial vector, Anopheles gambiae [35], in its local ecological community and to assess the effects of different control strategies on non-target organisms.”
- Line 228: "Neuroptera had the lowest completeness at 13.5%" - mention the sample size for this order.
We now include this (lines 242-243) “Neuroptera had the lowest completeness at 13.5% (137 samples), while Trichoptera had the highest at 71.1% (571 samples).”
- Line 302: "β = -1.92, p >0.05" - report the exact p-value.
Now updated

Editorial Manager®

Cookie Preference Center

We use cookies which are necessary to make our site work. We may also use additional cookies to analyze, improve and personalize our content and your digital experience. For more information, see our Cookie Policy.

You may choose not to allow some types of cookies. However, blocking some types may impact your experience of our site and the services we are able to offer. See the different category headings below to find out more or change your settings.

Allow all

Manage Consent Preferences

Strictly Necessary Cookies

Always active

These cookies are necessary for the website to function and cannot be switched off in our systems. They are usually only set in response to actions made by you which amount to a request for services, such as setting your privacy preferences, logging in or filling in forms. You can set your browser to block or alert you about these cookies, but some parts of the site will not then work.

Functional Cookies

These cookies enable the website to provide enhanced functionality and personalisation. They may be set by us or by third party providers whose services we have added to our pages. If you do not allow these cookies then some or all of these services may not function properly.

Performance Cookies

These cookies allow us to count visits and traffic sources so we can measure and improve the performance of our site. They help us to know which pages are the most and least popular and see how visitors move around the site.

Targeting Cookies

These cookies may be set through our site by our advertising partners. They may be used by those companies to build a profile of your interests and show you relevant adverts on other sites. If you do not allow these cookies, you will experience less targeted advertising.

Cookie List

Clear

Apply Cancel
Consent Leg.Interest

Confirm my choices

Power
