# Supplementary material for: Characterizing a species-rich and understudied tropical insect fauna using DNA barcoding
Source: Gigascience. 2026 Mar 17;15:giag028. doi: 10.1093/gigascience/giag028 (PMC13108253; doi:10.1093/gigascience/giag028)
Supplement: giag028_Reviewer_1_Report_original_submission — Reviewer 1 -- 12/9/2025 [file giag028_reviewer_1_report_original_submission.pdf]

Reviewer Report

Title: Characterising a species-rich and understudied tropical insect fauna using DNA barcoding

Version: Original Submission **Date:** 12/9/2025

Reviewer name: Reviewer 1

Reviewer Comments to Author:

The manuscript "Characterising a species-rich and understudied tropical insect fauna using DNA Barcoding" by Hemprich-Bennett and co-authors provides DNA barcodes from 95,996 individuals sampled in Ghana using various trap systems. In total, 10,120 unique BINs were identified, including 4,939 that were newly generated. Most sampled taxa were Diptera, Coleoptera, and Lepidoptera. In addition, the authors compared the determined BINs with already published data at BOLD, revealing the greatest overlap in BIN sharing with South Africa.

In my eyes, the topic of this manuscript is interesting and for suitable for a publication in "GigaScience" that is focusing on "big data" research. The amount of new sequence data for arthropods, in particular insects, is awesome and represents an important step to assess the (molecular) biodiversity, or better species diversity, of a super diverse region which has hardly been studied so far. The authors use state-of-the-art methods to analyze their data including the BOLD database and BIN approach. However, there are some points that should be added or discussed in a broader context (see below). In addition, please find some specific comments made via sticky notes on the PDF file of the manuscript.

I feel that the authors should provide some more references on various topics, especially in the introduction but discussion, too.

It would be nice to present some maps, photos of the collection sites, the sampling devices as well as the samples themselves as part of the main manuscript, documenting the efforts that were taken.

A BIN does per se not represent a species, because the variability of the DNA barcode fragment and mitochondrial DNA in general can be affected by various effects, e.g., incomplete lineage sorting, Wolbachia infections (especially true for arthropods), phylogeographic events, hybridization, and others. As consequence, BIN sharing and splitting can be observed - and in fact such effects are more often found than expected. It is fully clear that such analysis cannot be done for the given dataset, but a discussion of these effects is important and has been lacking thus far.

What happened with the vouchers and DNA extracts? It is obvious that the collected specimens will include a high number of undescribed species, therefore the deposition of the voucher specimens is highly important.

In my eyes it would be interesting to provide a summary of the lengths of the barcodes that were studied. How many barcodes were complete with a length of 658 base pairs? How many were about 300 bp etc.? I think such analysis can be easily done and visualized.

Please find some other specific suggestions for corrections or additions made via notes on the document file of the manuscript.

Methods

Are the methods appropriate to the aims of the study, are they well described, and are necessary controls included? Choose an item.

Conclusions

Are the conclusions adequately supported by the data shown? Choose an item.

Reporting Standards

Does the manuscript adhere to the journal's guidelines on minimum standards of reporting? Choose an item.

Choose an item.

Statistics

Are you able to assess all statistics in the manuscript, including the appropriateness of statistical tests used? Choose an item.

Quality of Written English

Please indicate the quality of language in the manuscript: Choose an item.

Declaration of Competing Interests

Please complete a declaration of competing interests, considering the following questions:

- Have you in the past five years received reimbursements, fees, funding, or salary from an organisation that may in any way gain or lose financially from the publication of this manuscript, either now or in the future?
- Do you hold any stocks or shares in an organisation that may in any way gain or lose financially from the publication of this manuscript, either now or in the future?
- Do you hold or are you currently applying for any patents relating to the content of the manuscript?
- Have you received reimbursements, fees, funding, or salary from an organization that holds or has applied for patents relating to the content of the manuscript?
- Do you have any other financial competing interests?
- Do you have any non-financial competing interests in relation to this paper?

If you can answer no to all of the above, write 'I declare that I have no competing interests' below. If your reply is yes to any, please give details below.

I declare that I have no competing interests

I agree to the open peer review policy of the journal. I understand that my name will be included on my report to the authors and, if the manuscript is accepted for publication, my named report including any attachments I upload will be posted on the website along with the authors' responses. I agree for my report to be made available under an Open Access Creative Commons CC-BY license (http://creativecommons.org/licenses/by/4.0/). I understand that any comments which I do not wish to be included in my named report can be included as confidential comments to the editors, which will not be published.

Choose an item.

To further support our reviewers, we have joined with Publons, where you can gain additional credit to further highlight your hard work (see: https://publons.com/journal/530/gigascience). On publication of this paper, your review will be automatically added to Publons, you can then choose whether or not to claim your Publons credit. I understand this statement.

Yes Choose an item.
