# Supplementary material for: Characterizing a species-rich and understudied tropical insect fauna using DNA barcoding
Source: Gigascience. 2026 Mar 17;15:giag028. doi: 10.1093/gigascience/giag028 (PMC13108253; doi:10.1093/gigascience/giag028)
Supplement: giag028_Reviewer_2_Report_original_submission — Reviewer 2 -- 1/5/2026 [file giag028_reviewer_2_report_original_submission.pdf]

Reviewer Report

Title: Characterising a species-rich and understudied tropical insect fauna using DNA barcoding

Version: Original Submission **Date:** 1/5/2026

Reviewer name: Reviewer 2

Reviewer Comments to Author:

General Comments:
This manuscript presents an impressive and highly valuable study that significantly advances our understanding of tropical arthropod diversity in West Africa. The sampling effort is extraordinary (nearly 100,000 individuals sequenced), and the dataset generated more than doubles the number of Barcode Index Numbers (BINs) publicly available for the region. The study is well-designed, employing multiple complementary trap types to capture diverse components of the arthropod community. The analyses are generally robust and appropriate for the research questions. The public release of this large dataset is a major contribution that will undoubtedly stimulate further taxonomic and ecological research in understudied tropical regions. The manuscript is clearly written and well-structured. I am generally in favour of acceptance after minor revisions.
Specific Comments and Suggestions for Revision:
1. Visual Documentation of Methods
The manuscript would benefit from including representative photographs of each of the five trap types (Malaise, yellow pan, pitfall, Heath, CDC) as deployed in the field. This is particularly helpful for readers less familiar with entomological methods. Given potential space constraints in the main text, I recommend including these as a Supplementary Figure (e.g., a panel of five photos with concise captions). Please cite this figure in the Methods (Sampling) section.
2. Robustness of Community Composition Analyses.
The NMDS and PERMANOVA results convincingly show differences among trap types. However, the sequencing effort (and thus sample size) varied greatly among traps (e.g., Heath: 65,293 samples vs. CDC: 3,039 samples). Could the authors please clarify if the Bray-Curtis dissimilarity matrices used in these analyses were calculated on standardized or rarefied data to account for this large disparity in sample size? A brief note in the Methods (Data analyses) or figure legend would assure readers that the observed patterns are not primarily an artefact of sampling intensity.
The finding of significantly higher diurnal catches (individuals and BINs) in Malaise traps is interesting. The discussion briefly mentions variance in thermal conditions. Could the authors expand the Discussion (Diurnal activity patterns) to include other potential ecological or methodological explanations? For example, might this reflect true peaks in flight activity for dominant taxa (Diptera, Hymenoptera), or could it be influenced by trap visibility or wind patterns differing between day and night? A sentence or two of speculation would enrich the interpretation.
The authors transparently note that only 34 of 117 Malaise lots were fully sequenced and that spiders were removed from some analyses. In the Discussion, please add a short statement evaluating how these practical limitations might have influenced the key conclusions regarding trap complementarity

and overall community completeness. For instance, does the high rate of BIN accumulation in Malaise traps (Supplementary Figure 6) suggest that sequencing the remaining lots might have yielded many additional unique BINs, potentially altering the estimated contribution of this trap type?
3. Minor Editorial and Clarity Points:
Line 381: There is a typo: "more insFect individuals" should be "more insect individuals".
Figure 2 & 3 Citations in Text: The in-text citations for Figures 2 and 3 (e.g., lines 239, 274-277) are currently embedded in the legend descriptions copied from the PDF. These should be simplified to standard figure calls (e.g., "(Figure 2)", "(Figure 3A, B)") and the legend text removed from the main manuscript body.

Methods

Are the methods appropriate to the aims of the study, are they well described, and are necessary controls included? Choose an item.

Conclusions

Are the conclusions adequately supported by the data shown? Choose an item.

Reporting Standards

Does the manuscript adhere to the journal's guidelines on minimum standards of reporting? Choose an item.

Choose an item.

Statistics

Are you able to assess all statistics in the manuscript, including the appropriateness of statistical tests used? Choose an item.

Quality of Written English

Please indicate the quality of language in the manuscript: Choose an item.

Declaration of Competing Interests

Please complete a declaration of competing interests, considering the following questions:

- Have you in the past five years received reimbursements, fees, funding, or salary from an organisation that may in any way gain or lose financially from the publication of this manuscript, either now or in the future?
- Do you hold any stocks or shares in an organisation that may in any way gain or lose financially from the publication of this manuscript, either now or in the future?

- Do you hold or are you currently applying for any patents relating to the content of the manuscript?
- Have you received reimbursements, fees, funding, or salary from an organization that holds or has applied for patents relating to the content of the manuscript?
- Do you have any other financial competing interests?
- Do you have any non-financial competing interests in relation to this paper?

If you can answer no to all of the above, write 'I declare that I have no competing interests' below. If your reply is yes to any, please give details below.

I declare that I have no competing interests.

I agree to the open peer review policy of the journal. I understand that my name will be included on my report to the authors and, if the manuscript is accepted for publication, my named report including any attachments I upload will be posted on the website along with the authors' responses. I agree for my report to be made available under an Open Access Creative Commons CC-BY license (http://creativecommons.org/licenses/by/4.0/). I understand that any comments which I do not wish to be included in my named report can be included as confidential comments to the editors, which will not be published.

Choose an item.

To further support our reviewers, we have joined with Publons, where you can gain additional credit to further highlight your hard work (see: https://publons.com/journal/530/gigascience). On publication of this paper, your review will be automatically added to Publons, you can then choose whether or not to claim your Publons credit. I understand this statement.

Yes Choose an item.
